# Activation of peroxymonosulfate by metal (Fe, Mn, Cu and Ni) doping ordered mesoporous Co_3_O_4_ for the degradation of enrofloxacin

**DOI:** 10.1039/c7ra07841b

**Published:** 2018-01-09

**Authors:** Jing Deng, Chen Ya, Yongjian Ge, Yongqing Cheng, Yijing Chen, Mengyuan Xu, Hongyu Wang

**Affiliations:** College of Civil Engineering and Architecture, Zhejiang University of Technology 18 Chaowang Road, Xiacheng District Hangzhou 310014 China hywangzjut@163.com +86 571 88320180 +86 571 88320180

## Abstract

Various transition metals (Fe, Mn, Cu and Ni) were doped into ordered mesoporous Co_3_O_4_ to synthesize Co_3_O_4_-composite spinels. Their formation was evidenced by transmission electronic microscopy (TEM), X-ray diffraction (XRD) and Brunauer–Emmett–Teller (BET) analysis. It was found that Co_3_O_4_-composite spinels could efficiently activate peroxymonosulfate (PMS) to remove enrofloxacin (ENR) and the catalytic activity followed the order Co_3_O_4_–CuCo_2_O_4_ > Co_3_O_4_–CoMn_2_O_4_ > Co_3_O_4_–CoFe_2_O_4_ > Co_3_O_4_–NiCo_2_O_4_. Moreover, through the calculation of the specific apparent rate constant (*k*_sapp_), it can be proved that the Co and Cu ions had the best synergistic effect for PMS activation. The Co_3_O_4_-composite spinels presented a wide pH range for the activation of PMS, but strong acidic and alkaline conditions were detrimental to ENR removal. Higher reaction temperature could promote the PMS activation process. Sulfate radical was identified as the dominating reactive species in Co_3_O_4_-composite spinel/PMS systems through radical quenching experiments. Meanwhile, the probable mechanisms concerning Co_3_O_4_-composite spinel activated PMS were proposed.

## Abstract 2

Various transition metals (Fe, Mn, Cu and Ni) were doped into ordered mesoporous Co_3_O_4_ to synthesize Co_3_O_4_-composite spinels.

## Graphical abstract

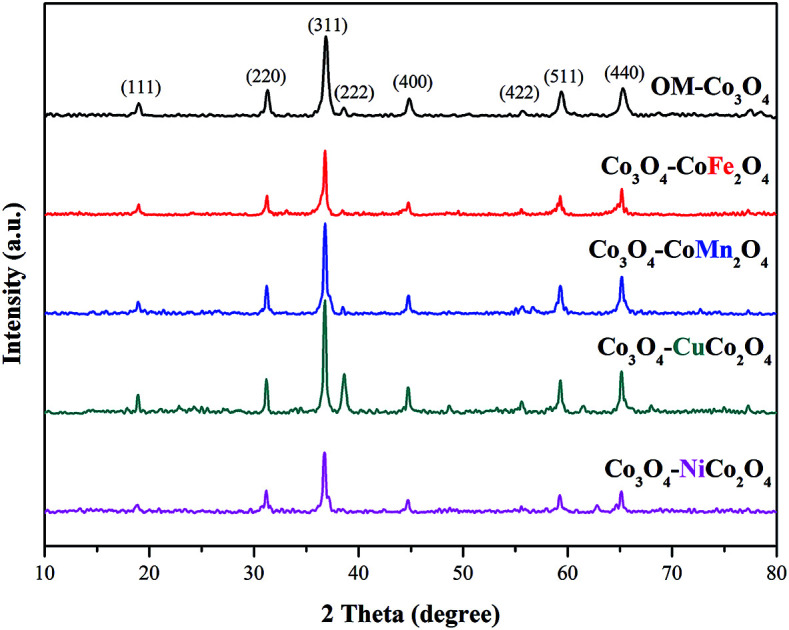

## 1. Introduction

During the past years, sulfate radical (SO_4_^−^˙) based advanced oxidation processes (SR-AOPs) have attracted an increasing interest among researchers owing to their great potential in degradation or even mineralization of recalcitrant organic pollutants.^1^ Compared with the hydroxyl radical (˙OH), SO_4_^−^˙ possesses a longer lifespan, higher independence of pH and higher selectivity of oxidation.^2,3^ Peroxymonosulfate (PMS), a precursor of SO_4_^−^˙, is deemed as a cost-effective and environmental-friendly oxidant.^4^ PMS remains stable in aqueous solution and barely decomposes into SO_4_^−^˙ by itself, but it can be activated to produce SO_4_^−^˙ by the use of UV, transition metals, and some nonmetal catalysts.^5–7^ Among different activation technologies, transition metals have attracted much attention due to their lower energy consumption and higher activation efficiency. Actually, many transition metal ions such as Co^2+^, Mn^2+^, Ni^2+^, Fe^2+^, Ru^3+^, Ce^3+^ and so forth, have been proved as qualified catalysts for PMS activation.^2^ Of note, Co^2+^ has been found to possess the highest reactivity.^8^ Unfortunately, the Co^2+^/PMS process is unfavorable in practical application because of the toxicity of Co^2+^.

In order to relieve the secondary pollution, heterogeneous cobalt-based catalysts have become a research hotspot. Anipsitakis *et al.* firstly employed Co_3_O_4_ to activate PMS and found Co_3_O_4_ presented an excellent catalytic behavior in the activation of PMS.^9^ Chen *et al.* successfully prepared nanoscale Co_3_O_4_ and tested its catalytic performance in PMS solution, results showed that 0.2 mM acid orange 7 (AO7) can be completely degraded within 30 min by 2 mM PMS in the presence of 0.5 g L^−1^ Co_3_O_4_.^10^ Pu *et al.* fabricated three types of Co_3_O_4_ using different metal organic frameworks, and found that all the Co_3_O_4_ exhibited outstanding catalytic activity and the difference in catalytic ability can be attributed to the difference in specific surface area.^11^ Consequently, Co_3_O_4_/PMS system is quite acceptable from the view of application due to the high activation efficiency and limitation of cobalt leaching. However, on the basis of the underlying threat of cobalt ions, it is essential to take measures to further limit the cobalt leakage during PMS activation.

It is reported that bimetallic oxides may be desirable catalysts to ease the conflict between catalytic performance and metal ions leaching, because intimate interactions between two metals can effectively suppress the leakage of metal ions, such as Fe–Co interactions in CoFe_2_O_4_.^2^ Moreover, bimetallic oxides are also prominent PMS activators. Su *et al.* synthesized a series of Co_*x*_Fe_3−*x*_O_4_ nanoparticles and found that the higher cobalt content in Co_*x*_Fe_3−*x*_O_4_ showed the higher catalytic activity towards PMS.^12^ The high catalytic behavior of CoFe_2_O_4_ was also illustrated in our previous study.^13^ Yao *et al.* reported that CoMn_2_O_4_ showed stronger catalytic activity than Co_3_O_4_, Mn_2_O_3_ and their physical mixture due to the synergistic effects of Co and Mn species.^14^ Similarly, CuCo_2_O_4_ also exhibited high catalytic performance and low metal leachability in PMS solution.^15^ In our previous research, order mesoporous Co_3_O_4_ (OM-Co_3_O_4_) was fabricated and showed superior catalytic ability toward PMS than its spinel counterpart, but the leakage of cobalt was up to 77.74 μg L^−1^ which was higher than conventional Co_3_O_4_ nanoparticles.^16^ Therefore, it can be reasonably speculated that cobalt leaching will reduce if some transition metals are doped to OM-Co_3_O_4_ to form mixed spinels with cobalt.

Herein, diverse transition metals (*i.e.*, Fe, Mn, Cu and Ni) was introduced into OM-Co_3_O_4_ to synthesized a series of Co_3_O_4_-composite spinels which were characterized by transmission electronic microscope (TEM), X-ray diffraction (XRD), Brunauer–Emmett–Teller (BET) and Zeta potential analysis. Due to the ubiquitous detection in aquatic environment,^17^ enrofloxacin (ENR) was selected as target pollutant in this study. The catalytic activities of as-prepared Co_3_O_4_-composite spinels were systematically compared through apparent rate constant, PMS consumption, intensity of electron paramagnetic resonance (EPR) signal and specific apparent rate constant. Moreover, the effects of initial pH and reaction temperature during PMS activation were also investigated. Finally, a possible mechanism of PMS activation was proposed through quenching tests. To be best of our knowledge, it is the first time to apply order mesoporous Co_3_O_4_-composite spinels as effective PMS activators for the control of organic pollutants.

## 2. Materials and methods

### 2.1 Chemicals

ENR, acetonitrile (HPLC grade), Oxone (KHSO5·0.5KHSO4·0.5K2SO4, PMS, KHSO5 ≥ 47%) and 5,5-dimethyl-1-pyrroline-*N*-oxide (DMPO) were obtained from Aladdin Biochemical Technology Co., Ltd. (Shanghai, China). The silica templates KIT-6 were purchased from Nanjing XFNANO Materials Tech Co., Ltd. (Jiangsu, China). Other chemical reagents were purchased from Sinopharm Chemical Reagent Co., Ltd. (Shanghai, China). Deionized water (18 MΩ cm) was produced from an Ulupure water purification system (Shanghai, China).

### 2.2 Catalysts preparation and characterization

OM-Co_3_O_4_ was synthesized using nanocasting route with KIT-6 as hard template, and the procedure was conducted as described before.^16^ For the preparation of Co_3_O_4_-composite spinels, 1.0 g OM-Co_3_O_4_ was dispersed in 2.5 mL ethanol of Fe(NO_3_)_3_·9H_2_O, Mn(NO_3_)_2_·4H_2_O, Cu(NO_3_)_2_·3H_2_O and Ni(NO_3_)_2_·6H_2_O, respectively, and Co/M molar ratio was controlled at 3. After magnetic stirring for 1 h, the mixture was dried overnight at 60 °C and then calcined at 450 °C for 5 h (the heating rate was set at 2 °C min^−1^). Finally, the obtained composite spinels were referenced as Co_3_O_4_–CoM_2_O_4_ (M = Fe, Mn) and Co_3_O_4_–MCo_2_O_4_ (M = Cu, Ni) which depended on the oxidation state of dopant.

The crystal structures of catalysts were characterized by X'Pert PRO diffractometer (PANalytical, Holland) with Cu Kα radiation. The morphologies and structures of Co_3_O_4_-composite spinels were observed using transmission electron microscopy (TEM) (Philips, Holland). N_2_ adsorption and desorption isotherms were measured using ASAP 2010 analyzer (Micromeritics, USA) at liquid nitrogen temperature (−196 °C). The pH at point of zero charge (pH_pzc_) was determined by Zetasizer Nano analyzer (Malvern, UK).

### 2.3 Catalytic experimental procedure

The catalytic degradation experiments were performed with a 100 mL ENR solution at 10 mg L^−1^ in 250 mL brown glass bottles, which were installed in a controlled temperature water bath stirring apparatus. In a typical run, specific amount of catalysts was added to ENR solution to receive adsorption–desorption equilibrium, followed by pH adjustment with H_2_SO_4_ and NaOH solution (100 mM) to ensure a desirable pH value after PMS addition. Subsequently, an appropriate amount of PMS was charged into the reaction solution to initiate experiment. At defined time intervals, 1 mL samples were collected and quenched by 0.1 mL Na_2_SO_3_ (100 mM). The resulting mixtures were immediately filtered by a 0.22 μm syringe filter for further analysis. All experiments were carried out in duplicates and the mean values were reported (with error bar).

### 2.4 Analytical methods

ENR concentrations were measured through a high-performance liquid chromatograph (HPLC, Agilent 1200, USA) with an Eclipse XDB-C18 column (5 μm particle, 150 × 4.5 mm), the concentrations were measured at *λ* = 278 nm using a mobile phase consisting of a mixture of acetonitrile and phosphoric acid (pH = 2.5) (v/v = 20 : 80) at a flow rate of 1.0 mL min^−1^. The PMS concentrations were measured by the method of Waclawek *et al.*^18^ EPR analysis were performed on a Bruker A300 spectrometer (Germany) with DMPO as a spin-trapping agent. The parameters of EPR spectrometer were center field was 3360.67 G, sweep width was 100 G, static field was 3310.66 G, microwave frequency was 9.42 GHz, microwave power was 2.03 mW, modulation amplitude was 1.0 G and sweep time was 30.72 s.

## 3. Results and discussion

### 3.1 Characterization

**Fig. 1:**
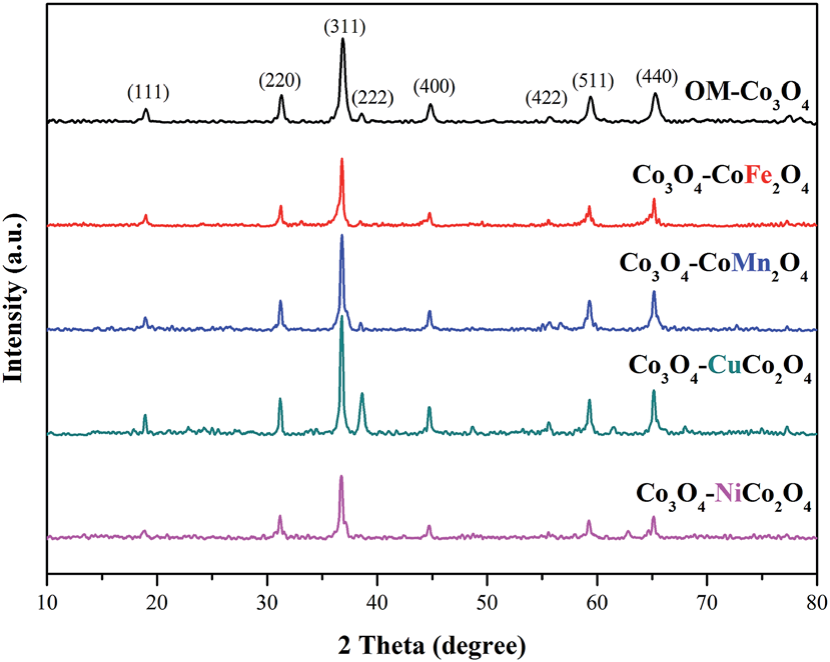
XRD patterns of OM-Co_3_O_4_ and Co_3_O_4_-composite spinels.

The crystalline phases of OM-Co_3_O_4_ and Co_3_O_4_-composite spinels were displayed in Fig. 1. It was worth noting that the precursors and OM-Co_3_O_4_ would result in the formation of composite spinels at high temperature. The lattice of Co_3_O_4_ would host other cations through the replacement of cobalt cations.^19^ However, no significant difference can be observed between OM-Co_3_O_4_ and Co_3_O_4_-composite spinels in Fig. 1. The well-defined diffraction peaks of 2*θ* = 19.00°, 31.27°, 36.85°, 38.54°, 44.81°, 55.66°, 59.36° and 65.24° were corresponded to (111), (220), (311), (222), (400), (422), (511) and (440), respectively. This might be ascribed to that the unit cell parameters of CoFe_2_O_4_, CoMn_2_O_4_, CuCo_2_O_4_ and NiCo_2_O_4_ were very close to that of Co_3_O_4_,^19^ thus these phases cannot be distinguished through XRD analysis. But according to Debye–Scherrer equation, the calculated mean crystallite sizes of OM-Co_3_O_4_, Co_3_O_4_–CoFe_2_O_4_, Co_3_O_4_–CoMn_2_O_4_, Co_3_O_4_–CuCo_2_O_4_ and Co_3_O_4_–NiCo_2_O_4_ were 17.43, 25.32, 22.87, 27.35 and 24.86 nm, respectively. Compared with OM-Co_3_O_4_, the increase of mean crystallite sizes in Co_3_O_4_-composite spinels indicated that the introduction of metal dopants destroyed original structure of OM-Co_3_O_4_.

**Fig. 2:**
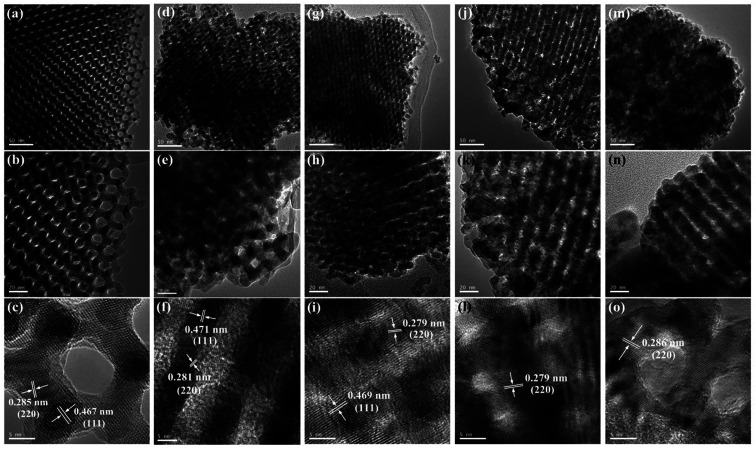
TEM and HR-TEM images of OM-Co_3_O_4_ and Co_3_O_4_-composite spinels. (a)–(c) OM-Co_3_O_4_; (d)–(f) Co_3_O_4_–CoFe_2_O_4_; (g)–(i) Co_3_O_4_–CoMn_2_O_4_; (j)–(l) Co_3_O_4_–CuCo_2_O_4_; (m)–(o) Co_3_O_4_–NiCo_2_O_4_.

The TEM and HR-TEM images of OM-Co_3_O_4_ and Co_3_O_4_-composite spinels were showed in Fig. 2. It can be clearly seen that OM-Co_3_O_4_ showed a highly ordered mesoporous structure, and the spacing distances between two fringes are 0.285 and 0.467 nm, which were in conformity with (220) and (111) planes, respectively. Obviously, after the introduction of metal dopants, ordered mesoporous structure was partly or completely destroyed, which may be attributed to the formation of Co_3_O_4_-composite spinels. The lattice fringes can be clearly observed in HR-TEM images, indicating that highly crystalline nature of Co_3_O_4_-composite spinels, which was corresponded to the strong and sharp diffraction peaks in XRD analysis. Similarly with OM-Co_3_O_4_, the spacing distances between two fringes in Co_3_O_4_–CoFe_2_O_4_ were 0.281 and 0.471 nm, corresponding to (220) and (111) planes, respectively. And that in Co_3_O_4_–CoMn_2_O_4_ were 0.279 and 0.469 nm, which were also assigned to (220) and (111) planes, respectively. However, as for Co_3_O_4_–CuCo_2_O_4_ and Co_3_O_4_–NiCo_2_O_4_, the (111) plane was not observed, and the lattice spacing of 0.275 and 0.286 nm was corresponded to (220) plane.

**Fig. 3:**
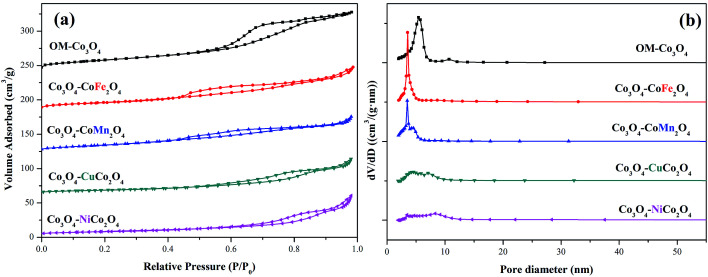
(a) Nitrogen adsorption–desorption isotherms of OM-Co_3_O_4_ and Co_3_O_4_-composite spinels and (b) their pore size distributions.

The surface areas and pore size distributions of OM-Co_3_O_4_ and composite materials were investigated by N_2_ adsorption–desorption isotherms. As shown in Fig. 3(a), all materials showed type IV isotherms, and the specific surface areas of OM-Co_3_O_4_, Co_3_O_4_–CoFe_2_O_4_, Co_3_O_4_–CoMn_2_O_4_, Co_3_O_4_–CuCo_2_O_4_ and Co_3_O_4_–NiCo_2_O_4_ were 66.91, 52.34, 50.92, 30.39 and 29.53 m^2^ g^−1^, respectively. The reduction of specific surface areas in Co_3_O_4_-composite spinels was ascribed to the deterioration of ordered mesoporous structure after the impregnation of metal cations, which was in accordance with the observation of TEM images. Moreover, compared with Co_3_O_4_–CoFe_2_O_4_ and Co_3_O_4_–CoMn_2_O_4_, the more significant decrease of specific surface areas in Co_3_O_4_–CuCo_2_O_4_ and Co_3_O_4_–NiCo_2_O_4_ may be related to the oxidation state of the doped metal ions. The formation of Co_3_O_4_–MCo_2_O_4_ consumed more Co_3_O_4_ than Co_3_O_4_–CoM_2_O_4_,^19^ thus the deterioration of ordered mesoporous structure in Co_3_O_4_–MCo_2_O_4_ was more significant than that of Co_3_O_4_–CoM_2_O_4_, which can also be observed from TEM images. The changes of pore volume and pore diameter also demonstrated the conclusion, as seen in the Fig. 3(b), the pore volume and pore diameter all followed the order of OM-Co_3_O_4_ > Co_3_O_4_–CoM_2_O_4_ > Co_3_O_4_–MCo_2_O_4_. The textural parameters of OM-Co_3_O_4_ and Co_3_O_4_-composite spinels were summarized in Table 1.

**Table 1:** Physicochemical properties of OM-Co_3_O_4_ and Co_3_O_4_-composite spinels

Samples	XRD	N_2_ adsorption–desorption			pH_pzc_
	Crystallite size (nm)	Surface area (m^2^ g^−1^)	Pore volume (cm^3^ g^−1^)	Pore diameter (nm)	
OM-Co_3_O_4_	17.43	66.91	0.135	8.08	5.85
Co_3_O_4_–CoFe_2_O_4_	25.32	52.34	0.105	7.19	4.21
Co_3_O_4_–CoMn_2_O_4_	22.87	50.92	0.094	6.32	3.93
Co_3_O_4_–CuCo_2_O_4_	27.35	30.39	0.083	5.91	4.76
Co_3_O_4_–NiCo_2_O_4_	24.86	29.53	0.086	6.21	5.37

**Fig. 4:**
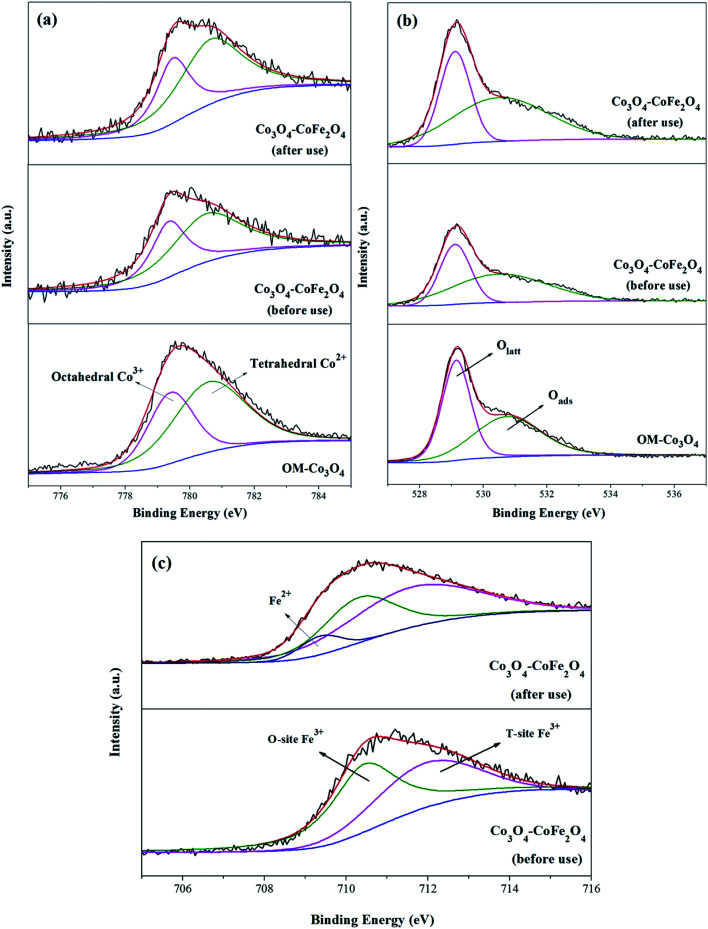
XPS survey spectrum of Co 2p_3/2_ (a), O 1s (b) and Fe 2p_3/2_ (c) for OM-Co_3_O_4_ and Co_3_O_4_–CoFe_2_O_4_.

XPS analysis can be used to determine the surface composition and chemical oxidation states of OM-Co_3_O_4_ and Co_3_O_4_-composite spinels. In XPS spectra of OM-Co_3_O_4_ (Fig. 4(a)), the sharp peak emerged at 779.6 eV was assignable to Co 2p_3/2_, which could be deconvoluted into octahedral Co^3+^ at 779.4 eV and tetrahedral Co^2+^ at 780.7 eV.^20^ The proportions of Co^2+^ and Co^3+^ were determined to be 63.01% and 36.99%, respectively. The O 1s envelope (Fig. 4(b)) could be deconvoluted into two parts, namely the lattice oxygen (O_latt_) at 529.2 eV and surface adsorbed oxygen (O_ads_) at 530.8 eV.^16^ Based on this deconvolution, the proportions of O_latt_ and O_ads_ were found to be 50.86% and 49.14%, respectively. After the doping of iron into OM-Co_3_O_4_, the content of Co^2+^ increased from 63.01% to 65.34%, which could be ascribed to the substitution of Co^3+^ with Fe^3+^ in OM-Co_3_O_4_. It was worth noting that the content of O_ads_ increased from 50.86% to 51.74%, which was conductive to the PMS activation.^4^ As seen in Fig. 4(c), the doped iron existed in the form of positive trivalent.

### 3.2 Catalytic activity of Co_3_O_4_-composite spinels

**Fig. 5:**
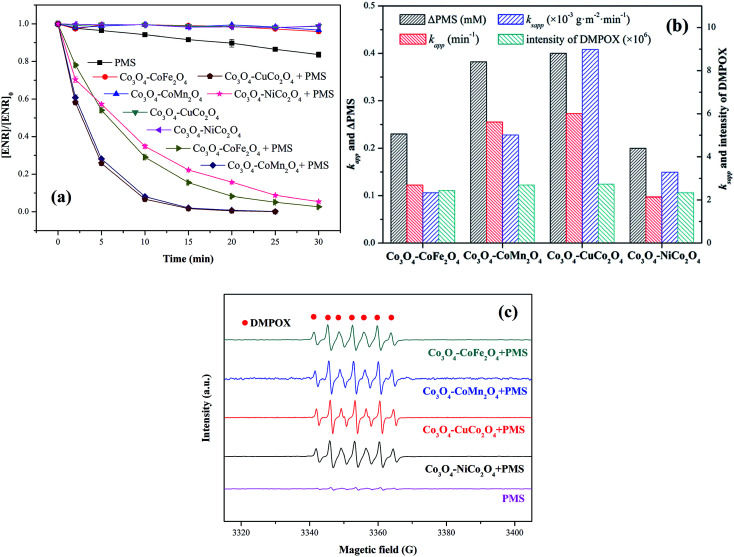
(a) ENR degradation in different systems; (b) values involved in different Co_3_O_4_-composite spinels/PMS systems; (c) EPR spectra in different composite spinels/PMS systems (DMPO = 25 mM). Experimental condition: [ENR] = 10 mg L^−1^, [catalyst] = 0.1 g L^−1^, [PMS] = 1 mM, pH_0_ = 6, *T* = 25 °C.

ENR removal in different systems was presented in Fig. 5(a). Adsorption tests showed that all the catalysts exerted a low efficiency in ENR adsorption, and the highest efficiency was received by Co_3_O_4_–CoFe_2_O_4_ with 3.22% of ENR adsorption within 30 min, which may be attributed to the largest specific surface area. Although PMS is a strong oxidizing agent with oxidation potential of 1.82 V,^21^ only 16.36% ENR could be removed by PMS in the absence of activator. However, the ENR degradation was greatly enhanced in the presence of both Co_3_O_4_-composite spinels and PMS. ENR can be completely removed in Co_3_O_4_–CuCo_2_O_4_/PMS and Co_3_O_4_–CoMn_2_O_4_/PMS systems, and the removal efficiencies were 96.37% and 94.56%, respectively. Furthermore, the ENR degradation well followed a pseudo-first-order kinetics pattern:1
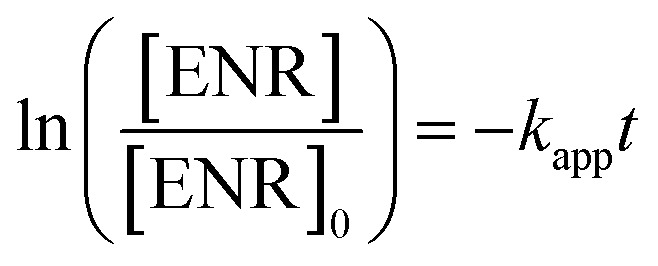
where [ENR]_0_ is the initial ENR concentration, [ENR] is the concentration of ENR at time *t*, and *k*_app_ is the apparent rate constant. As seen in Fig. 4(b), the fitting *k*_app_ for Co_3_O_4_–CoFe_2_O_4_, Co_3_O_4_–CoMn_2_O_4_, Co_3_O_4_–CuCo_2_O_4_ and Co_3_O_4_–NiCo_2_O_4_ are 0.122, 0.255, 0.273 and 0.097 min^−1^, respectively, which illustrated that the catalytic activity abided by the order of Co_3_O_4_–CuCo_2_O_4_ > Co_3_O_4_–CoMn_2_O_4_ > Co_3_O_4_–CoFe_2_O_4_ > Co_3_O_4_–NiCo_2_O_4_. In addition, Fig. 4(b) provided the PMS consumption during ENR oxidation. It can be seen that Co_3_O_4_–CuCo_2_O_4_ consumed the maximum PMS concentration (0.4 mM), which was 1.05, 1.74 and 2.00 times higher than Co_3_O_4_–CoMn_2_O_4_, Co_3_O_4_–CoFe_2_O_4_ and Co_3_O_4_–NiCo_2_O_4_, respectively. This result was also confirmed the sequence of catalytic activity of Co_3_O_4_-composite spinels.

EPR experiments were also performed for the comparison of the catalytic activity of Co_3_O_4_-composite spinels. As presented in EPR spectra (Fig. 5(c)), there was no distinctive EPR signal obtained by PMS alone. Nevertheless, simultaneous use of Co_3_O_4_-composite spinels and PMS could lead to obvious EPR signals. These EPR signals indicated the formation of 5,5-dimethyl-2-oxo-pyrroline-1-oxyl (DMPOX),^22,23^ which was ascribed to the fast activation of PMS and efficient oxidation of DMPO,^24^ also proofing the high catalytic performance of Co_3_O_4_-composite spinels. In addition, the signals of DMPOX caused by Co_3_O_4_–CuCo_2_O_4_/PMS and Co_3_O_4_–CoMn_2_O_4_/PMS systems were stronger than that caused by Co_3_O_4_–CoFe_2_O_4_ or Co_3_O_4_–NiCo_2_O_4_ activated PMS system (Fig. 5(b)), further authenticating the order of catalytic activity of Co_3_O_4_-composite spinels.

It was suggested that the catalytic performance of Co_3_O_4_-composite spinels not only depended on the specific surface area, but relied on dopant itself. In order to eliminate the difference in the specific surface area, the specific apparent rate constant *k*_sapp_ which defined as the ratio of *k*_app_ to the BET surface area was introduced:2
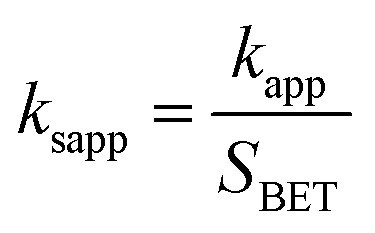
where *k*_sapp_ is the specific apparent rate constant, and *S*_BET_ is the specific surface area of composite spinels. As shown in Fig. 5(b), the *k*_sapp_ of Co_3_O_4_–CuCo_2_O_4_ (8.983 × 10^−3^ g (m^2^ min^−1^)) was still the highest one which was 3.85, 1.79, 2.73 times higher than that of Co_3_O_4_–CoFe_2_O_4_, Co_3_O_4_–CoMn_2_O_4_ and Co_3_O_4_–NiCo_2_O_4_, respectively, indicating that Co and Cu ions possessed the best synergistic effect for PMS activation.

### 3.3 Effect of initial pH

**Fig. 6:**
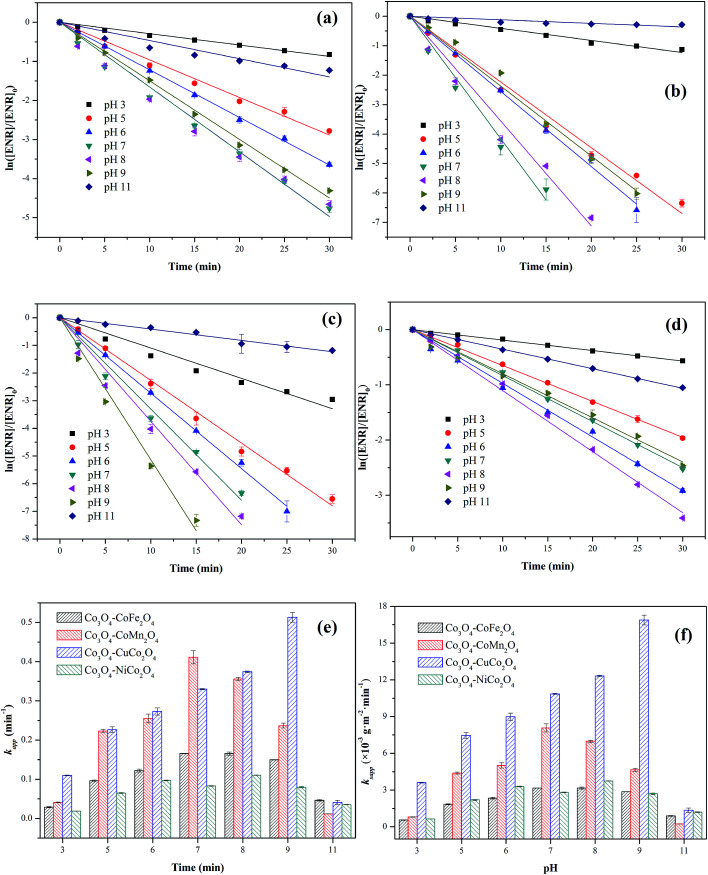
Effect of initial pH on ENR degradation in different Co_3_O_4_-composite spinels/PMS systems: (a) Co_3_O_4_–CoFe_2_O_4_; (b) Co_3_O_4_–CoMn_2_O_4_; (c) Co_3_O_4_–CuCo_2_O_4_; (d) Co_3_O_4_–NiCo_2_O_4_. Values involved in different pH and Co_3_O_4_-composite spinels/PMS systems: (e) *k*_app_; (f) *k*_sapp_. Experimental condition: [ENR] = 10 mg L^−1^, [catalyst] = 0.1 g L^−1^, [PMS] = 1 mM, *T* = 25 °C.

The influence of initial pH ranging from 3 to 11 on ENR degradation was investigated in the PMS activation process, and the results were displayed in Fig. 6. From Fig. 6(a)–(e), it can be seen that four Co_3_O_4_-composite spinels all showed a wide pH range for PMS oxidation and higher ENR removals were obtained in pH range of 5 to 9 while lower removals occurred at strong acidic and alkaline conditions. Similar results were also reported by the previous investigations, such as degradation of orange II in MnFe_2_O_4_/PMS process and removal of acetaminophen in Fe_3_O_4_/PMS system.^25,26^ The ENR degradation was significantly inhibited at strong acidic condition might be originated from the attachment of H^+^ to the peroxide bond (O–O) of PMS (eqn (3)) and the change of catalyst surface charge (eqn (4)), so that the interfacial repulsion would result in a weaker catalytic performance.^27^ The retardation of ENR removal at strong alkaline condition can be ascribed to the following reasons: (1) the increase of catalyst surface negative charges. The pH_pzc_ of Co_3_O_4_–CoFe_2_O_4_, Co_3_O_4_–CoMn_2_O_4_, Co_3_O_4_–CoCo_2_O_4_ and Co_3_O_4_–NiCo_2_O_4_ was 4.21, 3.93, 4.76 and 5.37, respectively (Table 1). The surface charges of catalysts were negative when solution pH was higher than pH_pzc_. Higher solution pH would cause higher amount of negative charges on catalyst surface, which could enhance the electrostatic repulsion between catalyst surface and PMS anions. Consequently, the catalytic performance decreased at strong alkaline condition. (2) The transform of dominant PMS species. Given that p*K*_a1_ of H_2_SO_5_ was less than 0 and p*K*_a2_ was 9.4, SO_5_^2−^ would replace HSO_5_^−^ and become dominant PMS species when solution pH was higher than 9.4. Compared with HSO_5_^−^ (*E*^0^(HSO_5_^−^/SO_4_^2−^) = 1.75 V), SO_5_^2−^ (*E*^0^(SO_5_^2−^/SO_4_^2−^) = 1.22 V) was less oxidative and more difficult to react.^28^ Additionally, SO_5_^2−^ could also lead to a stronger electrostatic repulsion between catalyst surface and PMS anions.

3 SO_2_ − O − O − H + H^+^ → SO_2_ − O − O − H_2_^+^

4 [Cat − OH] + H^+^ ↔ [Cat − OH_2_^+^]

The values of *k*_sapp_ were also calculated and the results were presented in Fig. 6(f). It could be more intuitionistic to compare the catalytic performances of four Co_3_O_4_-composite spinels in different conditions due to the elimination of difference in specific surface area. As shown in Fig. 5(f), Co_3_O_4_–CuCo_2_O_4_ maintained the highest *k*_sapp_ values with pH varied from 3 to 11, suggesting that Co and Cu species were the best combination for PMS activation among these four spinels. However, it should be noted that the lowest *k*_sapp_ value was showed by Co_3_O_4_–CoMn_2_O_4_ at pH 11 other than Co_3_O_4_–CoFe_2_O_4_ or Co_3_O_4_–NiCo_2_O_4_. This might be closely related to pH_pzc_ of catalysts, the pH_pzc_ of Co_3_O_4_–CoMn_2_O_4_ was 3.93 which was much lower than that of other three Co_3_O_4_-composite spinels. It manifested that Co_3_O_4_–CoMn_2_O_4_ could present lower performance at strong alkaline condition than others, thus Co_3_O_4_–CoMn_2_O_4_ possessed the highest *k*_sapp_ value.

### 3.4 Effect of temperature

**Fig. 7:**
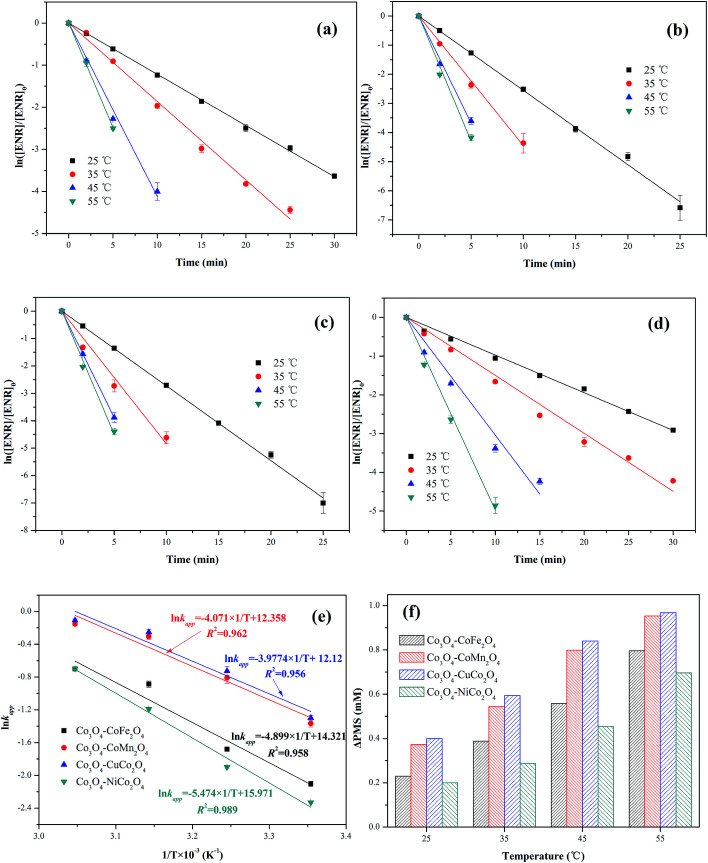
Effect of reaction temperature on ENR degradation in different Co_3_O_4_-composite spinels/PMS systems: (a) Co_3_O_4_–CoFe_2_O_4_; (b) Co_3_O_4_–CoMn_2_O_4_; (c) Co_3_O_4_–CuCo_2_O_4_; (d) Co_3_O_4_–NiCo_2_O_4_. Parameters involved in different reaction temperatures and Co_3_O_4_-composite spinels/PMS systems: (e) Arrhenius curves; (f) ΔPMS. Experimental condition: [ENR] = 10 mg L^−1^, [catalyst] = 0.1 g L^−1^, [PMS] = 1 mM, pH_0_ = 6.

The effect of reaction temperature (25, 35, 45 and 55 °C) on ENR removal in the process of PMS activation was studied. As displayed in Fig. 7(a–d), the ENR degradation in four Co_3_O_4_-composite spinels/PMS systems presented the similar trend, catalytic performances of Co_3_O_4_-composite spinels significantly increased with the increase of reaction temperature. As reaction temperature increased from 25 to 55 °C, *k*_app_ values of Co_3_O_4_–CoFe_2_O_4_, Co_3_O_4_–CoMn_2_O_4_, Co_3_O_4_–CuCo_2_O_4_ and Co_3_O_4_–NiCo_2_O_4_ increased from 0.122, 0.255, 0.273, 0.097 min^−1^ to 0.496, 0.872, 0.898, 0.498 min^−1^, respectively. This result may be due to the fact that higher reaction temperature simplified the rupture of O–O bond and generation of SO_4_^−^˙.^29,30^ In addition, higher reaction temperature was beneficial for reactant molecules to overcome activation energy barrier.^17^ The activation energy (*E*_a_) could be determined by plotting ln *k*_app_ against 1/T based on Arrhenius equation (Fig. 7(e)). The obtained *E*_a_ values were 40.73, 33.85, 33.07 and 45.51 kJ mol^−1^ in Co_3_O_4_–CoFe_2_O_4_, Co_3_O_4_–CoMn_2_O_4_, Co_3_O_4_–CuCo_2_O_4_ and Co_3_O_4_–NiCo_2_O_4_ activated PMS systems, respectively. The lower *E*_a_ value signified the higher catalytic reactivity, and the order of *E*_a_ was well corresponded to the sequence of catalytic activity. Moreover, all the *E*_a_ values were much higher than that of the diffusion-controlled reactions, which usually ranged from 10 to 13 kJ mol^−1^.^31^ This implied that the apparent reaction rate for ENR removal during Co_3_O_4_-composite spinels activated PMS processes was dominated by the rate of intrinsic chemical reactions on the catalyst surface. It was reported that out-sphere interactions were usually diffusion-controlled reactions, thus PMS activation by Co_3_O_4_-composite spinels was most likely an inner-sphere electron-transfer process.^32^ The consumption of PMS during ENR oxidation processes was monitored (Fig. 7(f)). Similar with the trend of *k*_app_, PMS consumption also increased as the reaction temperature increased. The PMS consumption caused by Co_3_O_4_–CoFe_2_O_4_, Co_3_O_4_–CoMn_2_O_4_, Co_3_O_4_–CuCo_2_O_4_ and Co_3_O_4_–NiCo_2_O_4_ increased from 0.230, 0.372, 0.400 and 0.200 mM to 0.796, 0.954, 0.968 and 0.696 mM with the reaction temperature increased from 25 to 55 °C, suggesting that higher reaction temperature was conducive to PMS activation and ENR degradation, which was well correspond to the conclusions by the observations of *k*_app_ values. In addition, the higher PMS consumption also reflected higher catalytic reactivity. From Fig. 7(f), the PMS consumption caused by Co_3_O_4_-composite spinels always showed the sequence of Co_3_O_4_–CuCo_2_O_4_ > Co_3_O_4_–CoMn_2_O_4_ > Co_3_O_4_–CoFe_2_O_4_ > Co_3_O_4_–NiCuFe_2_O_4_ even in different reaction temperatures, indicating that Co_3_O_4_–CuCo_2_O_4_ possessed the highest catalytic activity among the four Co_3_O_4_-composite spinels.

### 3.5 Radical identification and catalytic mechanism

Three different scavengers, *tert*-butyl alcohol (TBA), ethanol (EtOH) and phenol were employed to identify the dominant radical species in Co_3_O_4_-composite spinels/PMS systems. TBA can rapidly react with ˙OH (*k*_˙OH_ = 3.8–7.6 × 10^8^ M^−1^ s^−1^) but has a much lower reactivity with SO_4_^−^˙ (*k*_SO_4_^−^˙_ = 4–9.1 × 10^5^ M^−1^ s^−1^),^33^ and EtOH is a well scavenger for ˙OH and SO_4_^−^˙ (*k*_˙OH_ = 1.2–2.8 × 10^9^ M^−1^ s^−1^, *k*_SO_4_^−^˙_ = 1.6–7.7 × 10^7^ M^−1^ s^−1^).^34^ Phenol can also react with ˙OH and SO_4_^−^˙ at a high rate (*k*_˙OH_ = 6.6 × 10^9^ M^−1^ s^−1^, *k*_SO_4_^−^˙_ = 8.8 × 10^9^ M^−1^ s^−1^).^35^ In view of the difference in reaction rates, using TBA, EtOH and phenol as scavengers was a feasible program for the identification of primary active species.

**Fig. 8:**
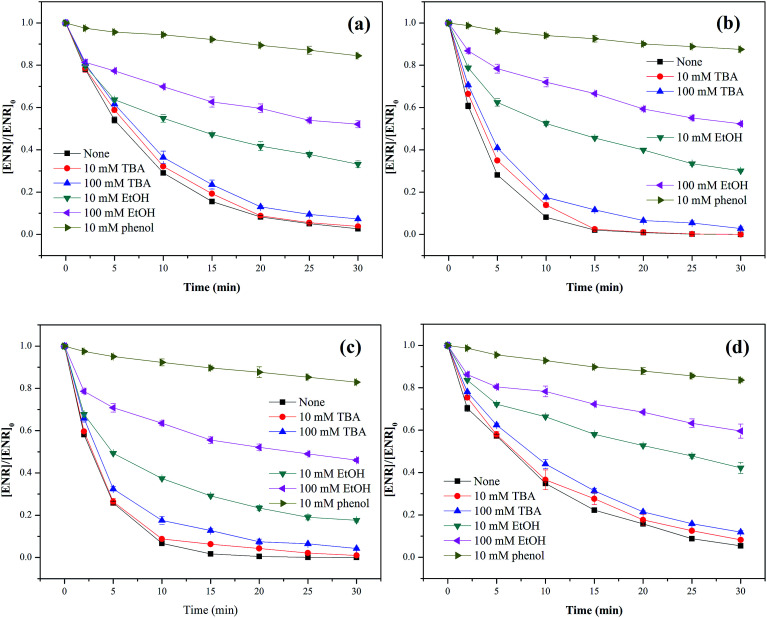
Effect of quenchers on ENR degradation in different Co_3_O_4_-composite spinels/PMS systems: (a) Co_3_O_4_–CoFe_2_O_4_; (b) Co_3_O_4_–CoMn_2_O_4_; (c) Co_3_O_4_–CuCo_2_O_4_; (d) Co_3_O_4_–NiCo_2_O_4_. Experimental condition: [ENR] = 10 mg L^−1^, [catalyst] = 0.1 g L^−1^, [PMS] = 1 mM, pH_0_ = 6, *T* = 25 °C.

As presented in Fig. 8(a–d), only a slight reduction of ENR removal could be obtained in the presence of 10 or 100 mM TBA, implying that ˙OH was involved in Co_3_O_4_-composite spinels activated PMS processes. With the addition of 10 mM EtOH, the ENR degradation was significantly inhibited and the removal efficiencies in Co_3_O_4_–CoFe_2_O_4_, Co_3_O_4_–CoMn_2_O_4_, Co_3_O_4_–CuCo_2_O_4_ and Co_3_O_4_–NiCo_2_O_4_ activated PMS processes were decreased from 97.37%, 100%, 100%, 94.56% to 66.84%, 69.92%, 82.42%, 57.81%, respectively. More addition of EtOH (100 mM) would cause further reduction of ENR removal efficiency with 47.90%, 47.70% 53.96% and 40.43%, respectively. In order to further confirm the contribution of SO_4_^−^˙, 10 mM phenol was introduced into solutions which resulted in a more significant inhibition to ENR removal, less than 18% of ENR could be decomposed in the four Co_3_O_4_-composite spinels/PMS processes. The quenching tests clearly suggested that SO_4_^−^˙ was the primary reactive species during PMS activation by Co_3_O_4_ composite spinels and ˙OH was also involved in these processes.

XPS analysis of Co_3_O_4_–CoFe_2_O_4_ before and after catalytic oxidation was also performed to illustrate the heterogeneous catalytic mechanism (Fig. 4). As shown in Fig. 4(a), before catalytic oxidation, the contents of Co^2+^ and Co^3+^ was determined to be 65.34% and 34.66%. After catalytic oxidation, the proportions of Co^2+^ and Co^3+^ were changed to 61.93% and 38.07%, respectively. The partial increase of Co^3+^ was ascribed to the electrons donating of Co^2+^ during the oxidation process. In the case of O 1s spectra (Fig. 4(b)), the content of O_latt_ decreased from 48.26% to 46.35%, and the proportion of O_ads_ increased from 51.74% to 53.65%. The increment of O_ads_ can be attributed to the generation of Co–OH or O_2_ adsorbed on the surface of Co_3_O_4_–CoFe_2_O_4_. It has been reported that ≡Co^2+^ − ^−^OH was the critical species for the generation of radicals during the process of PMS activation.^36^ Of note, the Fe 2p_3/2_ envelope could be deconvoluted into Fe^2+^ at 709.5 eV, which indicated that the redox reactions between Co and Fe were involved in the PMS activation.

Based on the results of quenching experiments and XPS analysis, the plausible mechanisms of Co_3_O_4_-composite spinels activated PMS were put forward. Taking Co_3_O_4_–CoFe_2_O_4_/PMS system as example, H_2_O molecules were firstly physically absorbed on the part of ≡Co^2+^ sites to generate ≡Co^2+^–^−^OH. Then, ≡Co^2+^ − ^−^OH would react with HSO_5_^−^ to form SO_4_^−^˙ after introduction of PMS (eqn (5)), and could regenerate through the reaction between formed ≡Co^3+^ − ^−^OH species and HSO_5_^−^ (eqn (6)). Similarly, ≡Fe^3+^ could also combine with dissociative adsorption of H_2_O molecules to form ≡Fe^3+^–^−^OH, which would transform to ≡Fe^2+^ − ^−^OH (eqn (7)) and generate SO_4_^−^˙ by reacting with HSO_5_^−^ (eqn (8)). In addition, due to the standard redox potential of ≡Co^3+^/≡Co^2+^ was 1.92 V,^8^ while the standard redox potential of ≡Fe^3+^/≡Fe^2+^ was 0.77 V,^37^ the reduction of ≡Co^3+^ by ≡Fe^2+^ was thermodynamically feasible (eqn (9)). The efficient regeneration of surface ≡Co^2+^ by this process may be able to remain the stability and high efficiency of Co_3_O_4_–CoFe_2_O_4_. Besides, ≡Co^2+^ or ≡Fe^2+^ on catalyst surface could also react with PMS to produce ˙OH (eqn (10) and (11)), which could also be generate by the transformation of SO_4_^−^˙ (eqn (12) and (13)). Co_3_O_4_–CoMn_2_O_4_, Co_3_O_4_–CuCo_2_O_4_ and Co_3_O_4_–NiCo_2_O_4_ activated PMS processes all presented the similar mechanism with Co_3_O_4_–CoFe_2_O_4_ activated PMS process, and the reactions which were involved in Co_3_O_4_-composite spinels/PMS systems were listed in Table 2.

**Table 2:** Reactions involved in composite spinels/PMS systems

Systems	Reactions	
Co_3_O_4_–CoFe_2_O_4_/PMS	≡Co^2+^ − ^−^OH + HSO_5_^−^ → ≡Co^3+^ − ^−^OH + SO_4_^−^˙ + OH^−^	(5)
	≡Co^3+^ − ^−^OH + HSO_5_^−^ → ≡Co^2+^ − ^−^OH + SO_5_^−^˙ + H^+^	(6)
	≡Fe^3+^ − ^−^OH + HSO_5_^−^ → ≡Fe^2+^ − ^−^OH + SO_5_^−^˙ + H^+^	(7)
	≡Fe^2+^ − ^−^OH + HSO_5_^−^ → ≡Fe^3+^ − ^−^OH + SO_4_^−^˙ + OH^−^	(8)
	≡Fe^2+^ + ≡Co^3+^ → ≡Fe^3+^ + ≡Co^2+^	(9)
	≡Co^2+^ + HSO_5_^−^ → ≡Co^3+^ + SO_4_^2−^ + ˙OH	(10)
	≡Fe^2+^ + HSO_5_^−^ → ≡Fe^3+^ + SO_4_^2−^ + ˙OH	(11)
	SO_4_^−^˙ + H_2_O → HSO_4_^−^ + ˙OH	(12)
	SO_4_^−^˙ + OH^−^ → SO_4_^2−^ + ˙OH	(13)
Co_3_O_4_–CoMn_2_O_4_/PMS	≡Co^2+^ − ^−^OH + HSO_5_^−^ → ≡Co^3+^ − ^−^OH + SO_4_^−^˙ + OH^−^	
	≡Co^3+^ − ^−^OH + HSO_5_^−^ → ≡Co^2+^ − ^−^OH + SO_5_^−^˙ + H^+^	
	≡Mn^3+^ − ^−^OH + HSO_5_^−^ → ≡Mn^2+^ − ^−^OH + SO_5_^−^˙ + H^+^	
	≡Mn^3+^ − ^−^OH + HSO_5_^−^ → ≡Mn^4+^ − ^−^OH + SO_4_^−^˙	
	≡Mn^2+^ − ^−^OH + HSO_5_^−^ → ≡Mn^3+^ − ^−^OH + SO_4_^−^˙	
	≡Mn^4+^ − ^−^OH + HSO_5_^−^ → ≡Mn^3+^ − ^−^OH + SO_5_^−^˙ + H^+^	
	≡Mn^2+^ + ≡Co^3+^ → ≡Mn^3+^ + ≡Co^2+^	
	≡Mn^3+^ + ≡Co^3+^ → ≡Mn^4+^ + ≡Co^2+^	
	≡Co^2+^ + HSO_5_^−^ → ≡Co^3+^ + SO_4_^2−^ + ˙OH	
	≡Mn^2+^ + HSO_5_^−^ → ≡Mn^3+^ + SO_4_^2−^ + ˙OH	
	SO_4_^−^˙ + H2O → HSO_4_^−^ + ˙OH	
	SO_4_^−^˙ + OH^−^ → SO_4_^2−^ + ˙OH	
Co_3_O_4_–CuCo_2_O_4_/PMS	≡Co^2+^ − ^−^OH + HSO_5_^−^ → ≡Co^3+^ − ^−^OH + SO_4_^−^˙ + OH^−^	
	≡Co^3+^ − ^−^OH + HSO_5_^−^ → ≡Co^2+^ − ^−^OH + SO_5_^−^˙ + H^+^	
	≡Cu^2+^ − ^−^OH + HSO_5_^−^ → ≡Cu^+^ − ^−^OH + SO_5_^−^˙ + H^+^	
	≡Cu^2+^ − ^−^OH + HSO_5_^−^ → ≡Cu^3+^ − ^−^OH + SO_4_^−^˙ + OH^−^	
	≡Cu^+^ − ^−^OH + HSO_5_^−^ → ≡Cu^2+^ − ^−^OH + SO_4_^−^˙ + OH^−^	
	≡Cu^3+^ − ^−^OH + HSO_5_^−^ → ≡Cu^2+^ − ^−^OH + SO_5_^−^˙ + H^+^	
	≡Cu^3+^ + ≡Co^2+^ → ≡Cu^2+^ + ≡Co^3+^	
	≡Cu^+^ + ≡Co^3+^ → ≡Cu^2+^ + ≡Co^2+^	
	≡Co^2+^ + HSO_5_^−^ → ≡Co^3+^ + SO_4_^2−^ + ˙OH	
	≡Cu^2+^ + HSO_5_^−^ → ≡ ^3+^ + SO_4_^2−^ + ˙OH	
	SO_4_^−^˙ + H_2_O → HSO_4_^−^ + ˙OH	
	SO_4_^−^˙ + OH^−^ → SO_4_^2−^ + ˙OH	
Co_3_O_4_–NiCo_2_O_4_/PMS	≡Co^2+^ − ^−^OH + HSO_5_^−^ → ≡Co^3+^ − ^−^OH + SO_4_^−^˙ + OH^−^	
	≡Co^3+^ − ^−^OH + HSO_5_^−^ → ≡Co^2+^ − ^−^OH + SO_5_^−^˙ + H^+^	
	≡Ni^2+^ − ^−^OH + HSO_5_^−^ → ≡Ni^3+^ − ^−^OH + SO_4_^−^˙ + OH^−^	
	≡Ni^3+^ − ^−^OH + HSO_5_^−^ → ≡Ni^2+^ − ^−^OH + SO_5_^−^˙ + H^+^	
	≡Co^2+^ + HSO_5_^−^ → ≡Co^3+^ + SO_4_^2−^ + ˙OH	
	≡Ni^2+^ + HSO_5_^−^ → ≡Ni^3+^ + SO_4_^2−^ + ˙OH	
	SO_4_^−^˙ + H_2_O → HSO_4_^−^ + ˙OH	
	SO_4_^−^˙ + OH^−^ → SO_4_^2−^ + ˙OH	

### 3.6 Reusability and stability of different Co_3_O_4_-composite spinels

**Fig. 9:**
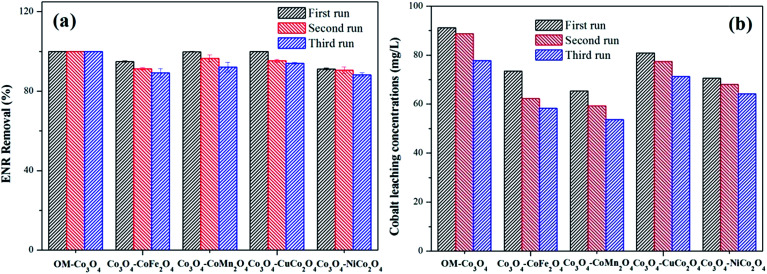
(a) Reusability of different Co_3_O_4_-composite spinels as catalyst for the degradation of ENR; (b) cobalt leaching concentrations in different Co_3_O_4_-composite spinels/PMS systems. Experimental condition: [ENR] = 10 mg L^−1^, [catalyst] = 0.1 g L^−1^, [PMS] = 1 mM, pH_0_ = 6, *T* = 25 °C, reaction time = 25 min.

The reusability of heterogeneous catalysts is an important indicator to assess the industrial application potential of heterogeneous catalysts. In this study, the reusability of different Co_3_O_4_-composite spinels was evaluated and the results were presented in Fig. 9(a). As shown, after being reused for three times, all the Co_3_O_4_-composite spinels still remained high catalytic activity toward PMS. Taking Co_3_O_4_–CoFe_2_O_4_ as example, after three consecutive runs, just a slight reduction in ENR removal was observed and their values were 94.89%, 91.32% and 89.27%, respectively. The slight loss of catalytic activity was mainly ascribed to the leaching of metal ions during the consecutive runs. Therefore, the stability of different Co_3_O_4_-composite spinels was further investigated and the results were reported in Fig. 9(b). As exhibited, the leaching of cobalt was clearly observed after each run. With the increase of cycle times, the leaching concentration decreased. Of note, after doped different transition metals into OM-Co_3_O_4_, the cobalt leakage can be effectively controlled, which was attributed to the intimate interactions between two metals.^2^ Therefore, Co_3_O_4_-composite spinels are ideal PMS activator for environmental remediation.

## 4. Conclusion

Co_3_O_4_-composite spinels were successfully synthesized through doping transition metals (Fe, Mn, Cu and Ni) to ordered mesoporous Co_3_O_4_. The obtained Co_3_O_4_-composite spinels all showed outstanding catalytic activity toward PMS. Co_3_O_4_–CuCo_2_O_4_ exhibited the highest catalytic performance in PMS solution, followed by Co_3_O_4_–CoMn_2_O_4_, Co_3_O_4_–CoFe_2_O_4_ and Co_3_O_4_–NiCo_2_O_4_. ENR degradation would be retarded in strong acidic and alkaline conditions, the improvement of reaction temperature could significantly accelerate ENR decomposition. Sulfate radical was confirmed to be the primary reactive species in Co_3_O_4_-composite spinels activated PMS processes and hydroxyl radical was also involved in these processes. The synergistic effect between two metals in Co_3_O_4_-composite spinels was the vital reason for the high catalytic reactivity. Co_3_O_4_-composite spinels displayed satisfactory reusability and doping different transition metals into OM-Co_3_O_4_ can effectively control the cobalt leaching. In consideration of cost and toxicity, Co_3_O_4_-composite spinels might have great potential in pollution control than OM-Co_3_O_4_.

## Conflicts of interest

No conflict of interest exists in the submission of this manuscript and manuscript is approved by all authors for publication.

## Supplementary Material
